# HLA-C Genotyping Reveals Haplotype C*07 as a Potential Biomarker of Late Psoriasis Onset in Moroccan Patients

**DOI:** 10.3390/cimb45020066

**Published:** 2023-01-25

**Authors:** Chaimaa Benlabsir, Myriam Riyad, Imane El Idrissi Saik, Hanaa Ettayebi, Oussama Aazzane, Kawtar Nassar, Soukaina Zaher, Siham Bennani, Brahim Admou, Samy Housbane, Khalid Sadki, Soumiya Chiheb, Hassan Fellah

**Affiliations:** 1 Laboratory of Cellular and Molecular Pathology, Research Team on Immunopathology of Infectious and Systemic Diseases, Faculty of Medicine and Pharmacy, Hassan II University of Casablanca, Casablanca 20000, Morocco; 2 Laboratory of Immunology, Ibn Rochd University Hospital Center, Casablanca 20000, Morocco; 3 Rheumatology Department, Ibn Rochd University Hospital Center, Casablanca 20000, Morocco; 4 HLA Typing Laboratory, Pasteur Institute of Morocco, Casablanca 20000, Morocco; 5 Laboratory of Immunology, Center of Clinical Research, University Hospital Mohammed VI, Faculty of Medicine and Pharmacy, Cadi Ayyad University, Marrakesh 40000, Morocco; 6 Medical Informatics Department, Faculty of Medicine and Pharmacy, Hassan II University of Casablanca, Casablanca 20000, Morocco; 7 Oral Biotechnology Laboratory, Fundamental Sciences Department, Faculty of Dental Medicine, Mohammed V University, Rabat 10000, Morocco; 8 Dermatology and Venerology Department, Ibn Rochd University Hospital Center, Casablanca 20000, Morocco

**Keywords:** psoriasis, HLA-C allele, methotrexate

## Abstract

Psoriasis still has an unknown etiology. Genetic predisposition shows the association between HLA-Cw6 allele and psoriasis. Although biotherapies have been proven effective in psoriasis treatment, methotrexate (MTX) is still used as a first-line systemic therapy due to its efficacy/affordability, but the differential response to MTX is mostly related to interindividual genetic variability and remains an issue. Our study aimed to analyze HLA-C allele frequencies in a sample of Moroccan psoriatic patients and assess the therapeutic response to MTX. Whole blood of 54 Moroccan psoriatic patients was collected and DNA was extracted. Patients’ HLA-C locus was genotyped by PCR-SSO. Results were analyzed with Luminex xMAP Technology and Match-it DNA Evolution 3.4. HLA-C typing results of 77 sex- and age-matched unrelated non-psoriatic healthy subjects were included. We observed no difference in the allelic distribution of HLA-C between patients and healthy controls, suggesting that none of the HLA-C alleles were significantly associated with psoriasis. Moreover, the HLA-C*07 allele was associated with a late age at disease onset (>30 years old) (*p* = 0.007). No statistically significant association was found between HLA-C allele expression and response to MTX, despite a higher frequency of HLA-C*06 in responders compared to non-responders. Thus, HLA-C*07 could be a biomarker of late psoriasis onset in the Moroccan population.

## 1. Introduction

Psoriasis is a systemic inflammatory dermatosis affecting 3–4% of the world’s population [1]. In 2015, a psoriasis working group in the Maghreb (North Africa) reported the following frequencies of new psoriasis cases: 15.04/1000 in Morocco, 10.26/1000 in Algeria, and 13.26/1000 in Tunisia [2]. In Morocco, general practitioners and dermatologists reported a similar prevalence of 0.11% in 2012 [3].

Psoriasis is characterized by a hyper-proliferation of keratinocytes resulting in a reddish scale and affecting several parts of the body, i.e., elbows, knees, scalp, and nails [4]. The disease is also associated with a reduced quality of life and shortened life expectancy, mostly due to its association with comorbidities such as cardiovascular diseases [5]. Even though its pathophysiology is poorly understood, the disease has been linked to genetic, immunological, and environmental factors [6]. The psoriasis susceptibility 1 (PSORS1) locus located on the major histocompatibility complex (HLA) on chromosome 6 has been identified as the most important genetic determinant in susceptibility studies [7]. In addition, Stuart et al. published a genome-wide association study (GWAS) of the genetic associations between psoriasis arthritis (PsA) and cutaneous psoriasis only (PsC). Five independent variants differentially associated with these two sub-phenotypes were found. Furthermore, this GWAS revealed a signal between HLA-C and HLA-B in the major histocompatibility complex (MHC) class I that showed the highest degree of differential association [8].

HLA-C is the most likely candidate gene for the PSORS1 locus, and the most significant association was described with HLA-CW*0602 [9,10]. The HLA-Cw6 isotype was strongly associated with severe psoriasis and early age of disease onset [11,12]. L. Puig et al. showed that 30% of psoriatic patients expressed the HLA-Cw6 allele, while its frequency in the general population was estimated at 10 to15% [13]. Other psoriasis susceptibility HLA alleles were identified, among them HLA-Cw1 in Asian populations [14]. A recent study identified the HLA-Cw7 allele as a possible biomarker of the early onset and severity of psoriasis in a Chinese population [15]. Brick et al. revealed respective frequencies of 14.7% and 24.4% for HLA-C06 and HLA-C07 alleles in 78 healthy subjects [16]. As far as we know, no research has been conducted on the HLA-C allelic frequencies in Moroccan psoriasis patients.

Disease severity at the time of diagnosis determines the appropriate treatment to prescribe, namely systemic therapy, phototherapy, and topical therapy. A small number of topical and systemic medications that are helpful for people with psoriasis are included in the World Health Organization’s (WHO) model list of essential medications and should be regarded as a basic necessity for all healthcare systems [17]. However, most targeted therapies remain unaffordable for the majority of patients, and methotrexate (MTX) remains the gold standard therapy. MTX (4-amino-N10methyl pteroylglutamic acid) is a folic acid antagonist that irreversibly and competitively inhibits the dihydrofolate reductase, a key enzyme in the production of folic acid. MTX is one of the earliest cancer chemotherapy agents still used to treat a variety of tumors [18]. It was initially introduced as a psoriasis treatment in 1958, and the US Food and Drug Administration later approved it in 1972 [19]. New updates about the care and management of psoriasis with different treatments including MTX were published in 2019 and 2020 as a collaboration between the National Psoriasis Foundation (NPF) and the American Academy of Dermatologists. In the special guide for systemic non-biologic therapies, several recommendations about MTX doses, folic acid supplementation, administration mode, and combined therapy of MTX with phototherapy were included. The need for the surveillance of renal and liver activity and cumulative doses of MTX was also highlighted [20]. Nevertheless, MTX is not fully effective since about one-third of treatment failure is reported due to numerous side effects, toxicity, and inter-individual genetic variability [21]. No data on MTX effects on psoriatic Moroccan patients are available.

In order to contribute to the better clinical management of psoriatic patients in Morocco, our study aimed to analyze HLA-C allele frequencies in a sample of Moroccan psoriatic patients and assess MTX therapeutic response.

## 2. Materials and Methods

### 2.1. Ethical Considerations

The present study adheres to Helsinki declaration guidelines and was approved by the local research ethics committee of Ibn Rochd University Hospital Center (Comité d’éthique du Centre Hospitalier Universitaire Ibn Rochd; Nb. 09/20; 24 April 2020). Patients provided written and informed consent.

### 2.2. Patients and Controls

Fifty-four patients who had received MTX for psoriasis treatment were recruited retrospectively and prospectively from the Dermatology-Venereology and Rheumatology departments of Ibn Rochd University Hospital Center in Casablanca. Patients were receiving weekly MTX intramuscularly (7–25 mg/per week). They also routinely received folic acid and a topical treatment alongside MTX. Some patients also received previous phototherapy treatment.

Demographic and clinical data were collected: patients’ age, gender, age of psoriasis onset, clinical phenotypes of psoriasis, nail involvement, concomitant psoriatic arthritis, comorbidities, risk factors, and their psoriasis family history. Furthermore, the baseline Psoriasis Area Severity Index (PASI) along with PASI at week 12 of MTX were also collected in order to assess the response to MTX, as well as side effects. Patients that needed to interrupt their treatment were also reported.

The average age of disease onset in our sample was 30.94 ± 19.13 years of age, and the median was 28 years of age. The age of disease onset in our sample showed a modal distribution (data not shown). Thus, patients were divided into two subgroups based on the age of psoriasis onset: early psoriasis onset (≤30 years old) and late psoriasis onset (>30 years old).

In order to analyze HLA-C allele frequencies in our patients, we used the HLA-C typing results of 77 non psoriatic healthy subjects, 48 from the HLA Typing Laboratory of Pasteur Institute of Morocco (Casablanca) and 29 from the Laboratory of Immunology, Center of Clinical Research, University Hospital Mohammed VI, Morocco (Marrakesh).

### 2.3. DNA Extraction

Whole blood was collected in EDTA tubes and genomic DNA was extracted using QuickGene DNA Whole Blood kit S (DB-S, KURABO, Kurashiki, Japan), according to the manufacturer’s instructions.

### 2.4. HLA-C Genotyping

HLA-C typing was performed with the kit LIFECODES HLA-C eRES SSO typing (IMMUCOR GTI Diagnostics, Waukesha, WI, USA), based on a sequence-specific oligonucleotide polymerase chain reaction (PCR-SSO) according to the manufacturer’s instructions. Typing results were analyzed with the Luminex xMAP Technology device and read by the software Match it DNA Evolution 3.4.

### 2.5. Efficacy Evaluation of Methotrexate Treatment

To assess the patient’s response to MTX, the baseline and 12-week PASI were calculated. Patients were designated as “non-responders” if they failed to show a clear improvement or had a PASI score of 50 or less (reducing their baseline PASI by 50%). Patients were classified as “responders” if they had a PASI of 75 (a reduction of 75 percent in PASI from the start of MTX therapy) or higher (PASI 90 and PASI 100).

### 2.6. Data Statistics and Analysis

A descriptive statistical analysis of demographic and clinical features was carried out. The following variables were taken into consideration: age, gender, age of psoriasis onset, clinical phenotypes of psoriasis, nail involvement, comorbidities, risk factors, psoriasis family history, the baseline PASI, PASI at week 12 of MTX treatment, MTX interruption, and side effects.

The comparison of demographic and clinical parameters between MTX responders and non-responders was subjected to univariate analysis by comparing means through Student’s *t*-test for quantitative variables, and comparing percentages using the Chi-squared test for qualitative variables.

HLA-C allele frequencies were determined by direct count. Comparisons of HLA frequencies between patients and healthy controls were performed using the Pearson x2 test with continuity correction. A *p*-value less than 0.05 was considered significant for all analyses. Data were analyzed by using SPSS Statistics software version 22 (IBM, Armonk, NY, USA).

## 3. Results

### 3.1. Demographic and Clinical Features of Psoriatic Patients

The age of our patients ranged from 11 to 72 years old, with a mean age of 42.28 ± 16.98 years old. There was a male predominance with a sex ratio M/F of 1.16.

The average age of disease onset was 30.94 ± 19.13; 30 patients (30/54; 55.56%) had an early age of disease onset (≤30 Years), and 24 (24/54; 44.44%) had a late age of disease onset (>30 Years).

The most common clinical phenotype was chronic plaque psoriasis diagnosed in 62.97% of patients (34/54), followed by erythrodermic psoriasis (14/54; 25.92%), and pustular psoriasis (6/54; 11.11%). Overall, 41 patients had skin involvement, 13 had concomitant joint involvement, and 18 patients (18/54; 33.33%) had nail involvement. Furthermore, eleven patients (11/54; 20.37%) reported a family history of psoriasis.

Three comorbidities were reported in our patients: diabetes (7 patients; 12.96%), obesity (5 patients; 9.25%), and hypertension (3 patients; 5.5%). Moreover, 3 risk factors were reported: stress (37/54; 68.51%), smoking (15/54; 27.77%), and alcohol consumption (5/54; 9.25%).

Regarding the follow-up and treatment of our patients, the mean baseline PASI was 30.31 ± 12.63. According to PASI score, most patients had severe psoriasis (53/54; 98.14%) and one patient had a moderate form of psoriasis.

In addition, PASI score at week 12 of MTX treatment was presented. On the whole, 9 psoriatic patients (9/54; 16.66%) had previous phototherapy and 13 patients (13/54; 24.07%) described tolerable side effects due to MTX such as nausea, headache, and vomiting so they could complete their treatment. Four patients (7.40%) interrupted their treatment because of liver and/or digestive toxicity. All demographic and clinical characteristics of our patients are shown in Table 1.

On the other hand, amongst the 54 patients, five were lost to follow-up and we had no information about their response to MTX treatment. Thus, the patients’ MTX response was analyzed for 49 patients that were classified as MTX responders or non-responders. For responders (N = 34), 15 patients (15/34; 44.11%) had a total lesion clearance and achieved PASI 100 by week 12 of MTX therapy, 9 patients (9/34; 26.47%) had a 90% reduction in baseline PASI, while a 75% PASI reduction was reached in 10 patients (10/34; 29.41%). For non-responders (N = 15), eight patients showed no improvement (8/15; 53.33) at all, of whom four (4/8) interrupted MTX therapy before week 12 due to hepatotoxicity or gastrointestinal toxicity, and finally seven patients (7/15; 46.66%) had a maximal PASI amelioration of 50%. Only mean follow-up PASI score was significantly associated with non-responders at week 12 (*p* = 0.002; OR = 0.558; 95% CI: 0.365–0.854) (Table 2).

### 3.2. HLA-C Alleles Expression

#### 3.2.1. Allelic Distribution of HLA-C Frequencies in Patients and Healthy Controls

**Figure 1:**
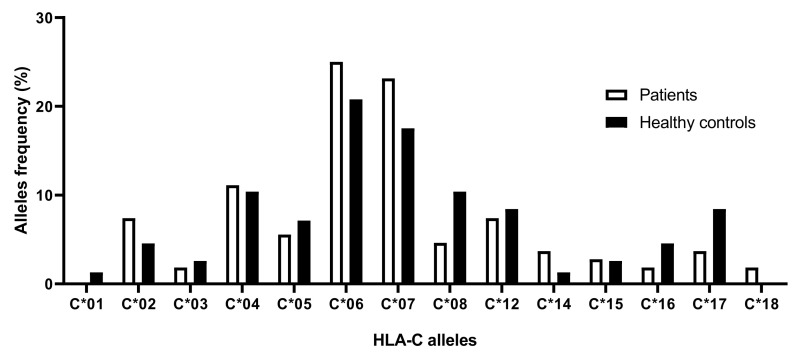
HLA-C allelic frequencies in psoriatic patients compared to healthy controls.

The analysis of HLA-C allele frequencies showed that alleles HLA-C*06 and HLA-C*07 displayed the highest values in both patients and controls, being 25% and 23.15%, respectively. They were also higher in patients than healthy controls (C*06 (25%) vs. C*06 (20.78%) and C*07 (23.15%) vs. C*07 (17.53%), respectively) as well as alleles HLA-C*02, HLA-C*04, and HLA-C*14, but with no overall statistical significance. HLA-C allele frequencies are shown in Figure 1.

#### 3.2.2. Allelic Distribution of HLA-C Frequencies According to the Age of Disease Onset

**Figure 2:**
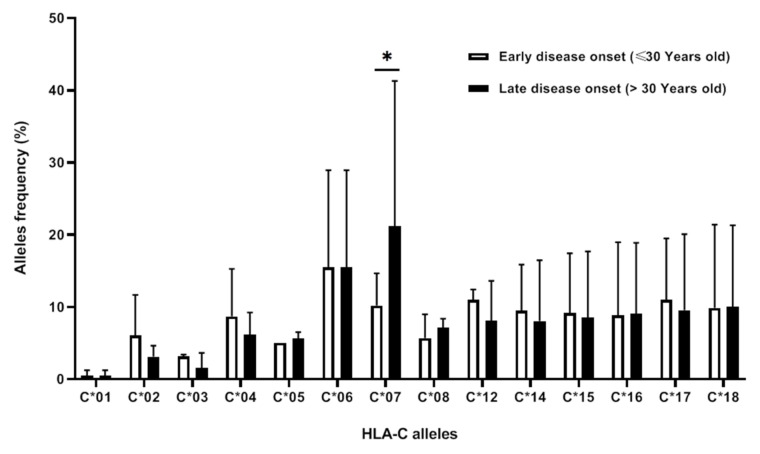
HLA-C allelic frequencies according to the age of disease onset. (*: *p* < 0.05 is statistically significant).

In our patients with late or early disease onset, only the HLA-C*07 allele was significantly more frequent in patients with late disease onset (*p* = 0.007; OR = 0.28; 95% CI: 0.11–0.73). The results are shown in Figure 2.

#### 3.2.3. Allelic Distribution of HLA-C Frequencies According to Psoriasis Clinical Phenotypes

**Figure 3:**
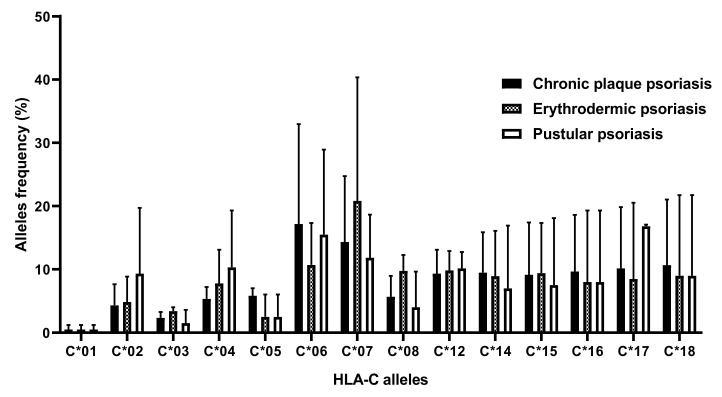
HLA-C allelic frequencies according to psoriasis clinical phenotypes.

HLA-C*06 and HLA-C*07 allele frequencies were the highest in the three psoriasis clinical phenotypes. However, no significant association was found between these phenotypes and any of the HLA-C alleles. Results are shown in Figure 3.

#### 3.2.4. Allelic Distribution of HLA-C Frequencies in Responders and Non-Responders to MTX

**Figure 4:**
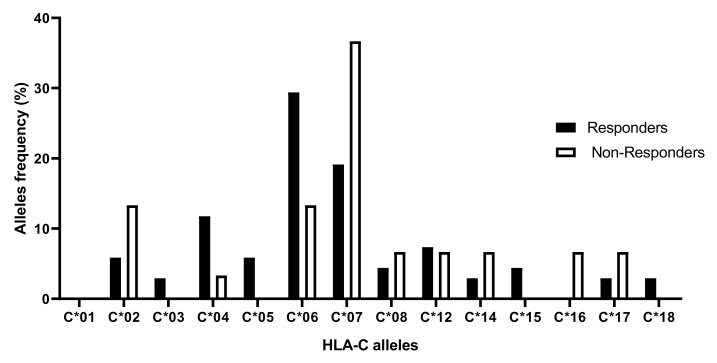
HLA-C allelic frequencies in responders and non-responders to MTX.

Comparison of HLA-C allele frequencies in responders and non-responders to MTX showed the highest allele frequencies for the HLA-C*06 allele in responders (C*06 (29.41%)) and HLA-C*07 allele in non-responders (C*07 (36.67%)). Furthermore, some HLA-C alleles were not expressed in non-responders (HLA-C*03, HLA-C*05, HLA-C*15, and HLA-C*18) while one was not expressed in responders (HLA-C*16). Overall, our results are not statistically significant (Figure 4).

## 4. Discussion

The WHO states that psoriasis is a widespread chronic non-communicable skin disease (NCD) with no known cause or treatment, negatively affecting the lives of patients. With a reported prevalence varying from 0.09 percent to 11.43 percent, psoriasis is a global issue that affects at least 100 million people of all ages worldwide. The unpredictable course of symptoms, the numerous external triggers, and the major comorbidities, such as psoriatic arthritis, cardiovascular diseases, metabolic syndrome, inflammatory bowel disease, and depression, were emphasized by the WHO [17]. Psoriatic arthritis is the most common concomitant inflammatory arthropathy, affecting 10 to 40% of psoriatic patients [22]. A GWAS was carried out on PsA patients, along with a meta-analysis of six other GWAS studies performed on PsA and PsC in order to distinguish pathogenic similarities between PsA and PsC. Thus, 10 regions had significant genome-wide (GW) associations with PsA, and 11 with PsC: IFNLR1, IFIH1, and NFKBIA for PsA, and TNFRSF9, LCE3C/B, TRAF3IP2, IL23A, and NFKBIA for PsC, respectively. Three of the identified psoriasis risk variants had a stronger association with PsC than PsA (rs12189871 near HLA-C, rs4908742 near TNFRSF9, rs10888503 near LCE3A), whereas two had a stronger association with PsA than PsC (rs12044149 near IL23R, rs9321623 near TNFAIP3) [8]. In Morocco, psoriatic diseases (psoriasis and psoriasis arthritis) are still misdiagnosed. As a result, psoriasis prevalence and incidence are only roughly estimated. This lack of awareness leads to late diagnosis and poor clinical management, resulting in disease evolution towards severe forms, which may explain their predominance in our patients (98.14%) [3].

PSOR1, located in the HLA-C gene, has been accepted as the major susceptibility gene linked to psoriasis [23]. Thus, our first aim was to analyze HLA-C frequencies in Moroccan psoriatic patients diagnosed and followed-up at University Hospital Center of Casablanca. Our results show no significant difference in HLA-C allelic frequencies between patients and healthy controls, suggesting that none of the HLA-C alleles were associated with psoriasis in our patients’ sample. However, HLA-C*06 and HLA-C*07 were predominant both in patients and controls, while HLA-C*01 and HLA-C*18 were not expressed in psoriatic patients and healthy controls, respectively. In addition, psoriasis has been reported to affect both genders equally [24]. In our sample, a slight male predominance (53.70%) was found, but was not statistically significant. Other studies also reported a male predominance, highlighting the need for further investigation emphasizing the impact of gender on the genetics of psoriasis [17,25,26].

Psoriasis is known as a T-cell-mediated autoimmune disease [27] besides genetic factors linked to psoriasis susceptibility. The HLA-Cw6 allele in PSOR1 region has been associated with psoriasis in several ethnic groups and geographical regions [28,29,30,31], along with early disease onset [32] and severe psoriasis forms [33]. A 1985 study showed an association between HLA-Cw6 and clinical family history. Furthermore, based on these findings, two psoriasis forms were defined: type I psoriasis characterized by an early disease onset before 40 years of age and HLA associations, and type II psoriasis beginning after 40 years of age and no HLA associations [12]. To our knowledge, this bimodal classification has not been confirmed further and no clear association with an average age of onset was found [32,34]. Our results show that the HLA-C*07 allele was significantly associated with late age of disease onset (*p* = 0.007), suggesting it could be a biomarker for late psoriasis onset, at least in Moroccan patients. Only a few studies reported the correlation between HLA-C*07 and psoriasis. According to some, there is no association between HLA-Cw*07 and psoriatic disease, and psoriatic arthritis patients are less likely to be HLA-Cw*07 carriers [35,36]. A positive correlation between psoriatic spondylitis and HLA-Cw*0702 was discovered in a Spanish study, and HLA-Cw*0701 was reported to be under-represented in PsA patients compared to controls [37]. Chinese psoriatic patients in Taiwan also had an increased frequency of HLA-Cw*0702 compared to a healthy population [38]. Amongst psoriasis risk-related leukocyte antigens, HLA-C*07:01, C*07:02, and B*27 utilize the same anchor residues with HLA-C*06:02, have overlapping peptide-binding properties, and belong to the same HLA supertype. Furthermore, the HLA-C*07:01 allele has been significantly associated with HLA-B alleles in several autoimmune and immune-mediated inflammatory diseases [39]. T cell receptors (TCR) have also shown abnormal activity in psoriasis. The relationship between the diversity of TCRs and MHC in psoriatic patients was investigated and it was shown that HLA-C*07:02 was positively correlated with the abundance of TCR, suggesting that people with the HLA-C*07:02 haplotype have high immunological activity that may contribute to the development of psoriasis [15].

Our sample of psoriatic patients was clinically heterogeneous (Table 1) and three types of psoriasis were diagnosed: chronic plaque psoriasis (psoriasis vulgaris), erythrodermic psoriasis, and pustular psoriasis. These 54 patients were treated with MTX, including the six patients presenting with pustular psoriasis (6/54). This latter phenotype is considered one of the psoriasis variants and one of the less frequent, according to the WHO [17]. Moreover, chronic plaque psoriasis was our patients’ most frequent clinical phenotype (62.97%), in agreement with earlier reports [34,40,41]. However, no association between HLA-C alleles and disease severity could be assessed probably because of this clinical heterogeneity and because most of them (53/54) presented severe psoriasis (53/54).

Furthermore, several pharmacogenetic studies have proven the association between gene expression and the response to systemic treatments and targeted therapies in psoriasis [33,42]. In Morocco, MTX is also the first-line therapy, and remains the treatment of choice with the best cost-effectiveness ratio [43]. The association between a good response to MTX and the expression of HLA-C alleles has been highlighted, especially HLA-Cw6. Indhumathi et al. reported that South Indian psoriatic patients expressing HLA-Cw6 had a better response to MTX [44]. Instead, our results show no significant association between HLA-C alleles and response to MTX probably due to our small sample size and the predominant severe forms. In addition, in our sample, demographic and clinical features were not associated with a good or bad response to MTX therapy, and none of the highly expressed alleles were correlated with a better response to MTX.

Despite the fact that genetic involvement has been proven in psoriasis, the trigger is yet unknown. Another important area of study that needs to be further explored involves the immune system’s contribution to the development of psoriasis. Several investigations have suggested that structural proteins have antigenic activities, and self-DNA complexes with Cathelicidin (LL-37) have lately been proposed as auto-antigens in some psoriasis cases [27]. Furthermore, environmental variables such as infection, stress, excess body weight, drugs, cigarette smoking, alcohol consumption, and even the weather and climate are known to cause and exacerbate psoriasis in some people [45]. Psoriasis has also been associated with a number of bacterial, viral, and even fungal illnesses [46,47].

Finally, we are aware of two main limitations of our study. Firstly, the low number of patients included in our sample may be explained by the misdiagnosis of the disease in Morocco due in part to the lack of patients’ awareness, leading to late diagnosis, but also to the patients lost to follow-up. The other limitation of our study lies in the heterogeneity of the psoriasis clinical phenotypes described in our patients that may explain the lack of association between HLA-C alleles and disease severity; MTX treatment outcome could also be assessed.

## 5. Conclusions

Our results show that the HLA-Cw7 allele was significantly associated with late psoriasis onset, suggesting it could be a biomarker for late disease onset, at least in Moroccan patients. Regarding the MTX treatment outcome of our patients, no association was found between HLA-C alleles and the response to MTX. HLA-C gene polymorphisms have been related to ethnic differences; thus, other genetic and environmental factors may play a role in psoriasis susceptibility and severity. However, additional genotyping is needed to confirm our findings. Other MTX response-related genes could be investigated in order to look for a biomarker of MTX response and improve the clinical management of our patients. Importantly, patients presenting with psoriasis are at higher risk of developing cardiovascular and other NCDs, but the disease can also affect mental health and may lead to significant social stigma. Currently used clinical outcome parameters need to be improved to assess the severity of psoriasis and the disease’s impact on quality of life, and used by health-care providers and dermatologists.

## Figures and Tables

**Table 1:** Demographic and clinical features of the 54 psoriatic patients.

Variables	Patients (N = 54)
**Demographic features**	
**- Mean age, years old (SD)**	42.28 (16.98)
**- Gender**	
Male	29 (53.70%)
Female	25 (46.30%)
Sex ratio (M/F)	1.16
**Clinical features**	
**- Age of disease onset, years old (SD)**	30.94 (19.13)
Early disease onset (≤30 Years)	30 (55.56%)
Late disease onset (>30 Years)	24 (44.44%)
**- Clinical phenotypes of psoriasis**	
Chronic plaque psoriasis (psoriasis vulgaris)	34 (62.97%)
Erythrodermic psoriasis	14 (25.92%)
Pustular psoriasis	6 (11.11%)
**- Nail involvement**	18 (33.33%)
**- Concomitant psoriasis arthritis**	13 (24.07%)
**- Comorbidities**	
Diabetes	7 (12.96%)
Obesity	5 (9.25%)
Hypertension	3 (5.50%)
**- Risk factors**	
Stress	37 (68.51%)
Smoking	15 (27.77%)
Alcohol intake	5 (9.25%)
**- Baseline PASI**	
Mean baseline PASI (SD)	30.31 (12.63)
Moderate Psoriasis (PASI 5–10)	1 (1.85%)
Severe Psoriasis (PASI > 10)	53 (98.14%)
**- PASI Score at week 12 of MTX**	
Mean PASI at week 12 of MTX (SD)	8.17 (12.97)
No improvement	8 (16.32%)
PASI 50	7 (14.28%)
PASI 75	10 (20.40%)
PASI 90	9 (18.36%)
PASI 100	15 (30.61%)
**- Tolerable side effects due to MTX**	13 (24.07%)
**- MTX interruption due to liver and/or digestive toxicity**	4 (7.40%)

**Table 2:** Demographic and clinical characteristics in responders and non-responders to MTX.

Variables	Responders (N = 34)	Non-Responders (N = 15)	*p*-Value
**Demographic features**			
**- Mean age, years old (SD)**	40.23 (17.80)	44 (16.46)	0.485
**- Gender**			
Male	18	8	0.980
Female	16	7	
Sex ratio (M/F)	1.125	1.14	
**Clinical features**			
**- Clinical phenotypes of psoriasis**			
Chronic plaque psoriasis (psoriasis vulgaris)	23 (67.60%)	7 (46.70%)	0.345
Erythrodermic psoriasis	8 (23.50%)	5 (33.33%)	
Pustular psoriasis	3 (8.90%)	3 (20%)	
**- Nail involvement**	15 (44.11%)	1 (6.67%)	0.081
**- Concomitant psoriasis arthritis**	6 (17.60%)	7 (46.70%)	0.077
**- Age of disease onset, years old (SD)**	28 (18.05)	33.27 (12.29)	
Early disease onset (≤30 Years)	22 (64.70%)	7 (46.70%)	0.235
Late disease onset (>30 Years)	12 (35.30%)	8 (53.30%)	
**- Family history of psoriasis**	8 (23.50%)	1 (6.67%)	0.315
**- PASI score**			
Mean baseline PASI (SD)	29.74 (13.03)	33.33 (12.29)	0.765
Mean PASI at week 12 of MTX (SD)	2.75 (2.85)	23.55 (17.66)	**0.002**
**- Side effects due to MTX**	6 (17.64%)	7 (46.70%)	0.077

## Data Availability

No new data were created or analyzed in this study. Data sharing is not applicable to this article.
